# Economic Burden of Patients With Chronic Idiopathic Constipation in the USA Before and After Prucalopride Initiation

**DOI:** 10.1016/j.gastha.2025.100664

**Published:** 2025-03-24

**Authors:** Paul Feuerstadt, Mei Lu, Emi Terasawa, Brian Terreri, Shawn Du, Selina Pi, Ben Westermeyer, Rajeev Ayyagari, Anthony Lembo, Baharak Moshiree, Mena Boules, Brooks D. Cash

**Affiliations:** 1 Physicians Alliance of Connecticut Gastroenterology Center, Hamden, Connecticut; 2 Yale Division of Digestive Diseases, Yale School of Medicine, Yale University, New Haven, Connecticut; 3 Takeda Pharmaceuticals USA, Inc., Lexington, Massachusetts; 4 Analysis Group, Inc., New York, New York; 5 Analysis Group, Inc., Boston, Massachusetts; 6 Digestive Disease & Surgery Institute, Cleveland Clinic, Cleveland, Ohio; 7 Department of Medicine, Atrium Health Wake Forest University School of Medicine, Charlotte, North Carolina; 8 Division of Gastroenterology, Hepatology and Nutrition, University of Texas Health Science Center at Houston, Houston, Texas

**Keywords:** Constipation, Chronic Idiopathic Constipation, Prucalopride, Health Care Resource Utilization, Health Care Costs.

## Abstract

**Background and Aims:**

Chronic idiopathic constipation (CIC) is associated with substantial health care resource utilization (HCRU) and economic burden; however, real-world evidence on the impact of treatment initiation on HCRU and health care costs are limited. We evaluated HCRU and direct health care costs associated with prucalopride initiation in patients with CIC in the United States.

**Methods:**

Data were collected between January 1, 2015, and June 30, 2020, from the IBM MarketScan Commercial Claims and Encounters and Medicare Supplemental Databases for 690 adults with ≥ 1 prescription fill for prucalopride and ≥ 1 constipation-related diagnosis code. All-cause and constipation-related HCRU (outpatient, emergency room, and inpatient visits) for all patients, and pharmacy and medical health care costs for those aged 18–64 years were assessed 6 months before (baseline) and after (study period) prucalopride initiation. Subpopulations examined were patients with prior CIC medication use and males with CIC.

**Results:**

Compared with baseline, the mean number of any constipation-related outpatient visits (2.26 vs 1.52; *P* < .001) and the proportions of patients with these visits (82.6% vs 58.8%; *P* < .001) significantly decreased after prucalopride initiation. In 564 patients, total constipation-related health care costs significantly increased from baseline during the study period (mean: $1497 vs $2332; *P* < .001), primarily driven by increased pharmacy costs after prucalopride initiation ($621 vs $1751; *P* < .001). Constipation-related medical costs significantly decreased during the study period ($876 vs $580; *P* < .001). Some constipation-related HCRU and health care costs decreased after prucalopride initiation in patients with prior CIC medication use and males.

**Conclusion:**

In adults with CIC, constipation-related HCRU and medical costs decreased, while constipation-related health care costs and pharmacy costs increased 6 months after prucalopride initiation.

## Graphical abstract

**Figure:**
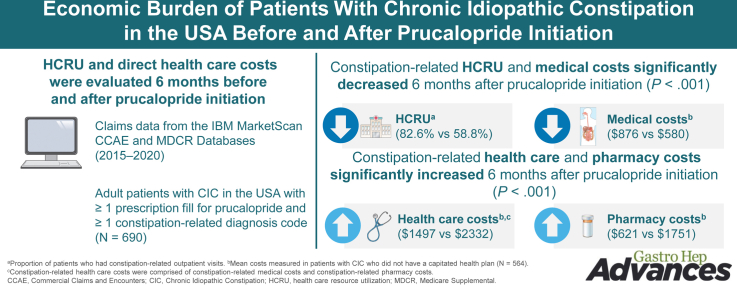

## Introduction

Chronic idiopathic constipation (CIC) is a functional bowel disorder characterized by infrequent or incomplete bowel movements.^1^^,^^2^ The global prevalence of CIC in patients has been estimated at 14%; CIC is generally higher in females and increases with age.^2^ In the United States, the prevalence of CIC is between 3.4% and 19.4%, affecting approximately 35 million adults.^2^^,^^3^ CIC is associated with a substantial health care and economic burden.^4^^,^^5^ The direct health care costs in patients with CIC in 2010 in the United States were estimated to be $9012 per year per patient^6^; these costs are likely to be much higher now. Patients with CIC also typically report a reduced health-related quality of life, have higher health care resource utilization (HCRU), and incur greater direct health care costs than patients without CIC.^7^, ^8^, ^9^

Current clinical guidelines from the American Gastroenterological Association (2023) for the management of chronic constipation recommend initiating therapy with readily available and relatively inexpensive therapies, such as over-the-counter (OTC) soluble fiber supplementation and/or laxatives.^10^ Pharmacologic or prescription agents should then be considered when symptoms do not respond to the aforementioned OTC treatments.^10^ According to a United States population-based survey carried out in 2018, 93.5% of individuals with constipation self-manage their symptoms using OTC therapeutics, with a minority of patients (1.3%) receiving prescription medication(s) only (while 5.2% of patients receive both OTC and prescription medications).^4^ One potential explanation for this is that nearly two-thirds of patients have never discussed their constipation symptoms with a health care provider.^4^

In the United States, prescription medications indicated for adults with CIC include secretagogues, such as lubiprostone, linaclotide, and plecanatide (approved in 2006, 2012, and 2017, respectively).^3^^,^^11^, ^12^, ^13^ Prucalopride, a prokinetic prescription medication, is a selective high-affinity serotonin type 4 receptor agonist^14^ that is indicated for the treatment of CIC in adults in the United States (approved in 2018) at a dosage of 2 mg once daily or 1 mg once daily in patients with severe renal impairment (creatine clearance < 30 mL/min).^14^ In an integrated analysis of 6 phase 3–4 trials, prucalopride was well tolerated and improved the frequency of spontaneous complete bowel movements per week compared with placebo over a 12-week period.^15^

Real-world data on the impact of initiating prescription treatment on HCRU and direct health care costs associated with CIC are currently limited.^16^, ^17^, ^18^ We therefore aimed to assess the real-world impact of prucalopride on HCRU and direct health care costs in patients with CIC in the United States.

## Methods

### Study Design and Population

This was an observational retrospective cohort study of prucalopride-treated patients based on insurance claims data (medical and pharmacy services) collected between January 1, 2015, and June 30, 2020, from the IBM MarketScan Commercial Claims and Encounters (CCAE) and Medicare Supplemental (MDCR) Databases.^19^ IBM MarketScan CCAE is a high-quality resource that comprises the combined claims of approximately 260 self-insured employers and 40 health plans in the United States, with all census regions represented. Since 1995, this database has captured approximately 240 million patients on insurance coverage; the database has a claims lag period of 9 months (ie, the time between the date of service on the claim and the date the payment is made). The MDCR Database includes information on the subset of Medicare beneficiaries who have supplemental insurance paid by their employers; in 2010, this represented approximately 14% of the 46 million retirees with Medicare benefits.^20^ The MDCR population includes active and retired employees and their Medicare-eligible dependents.^21^

Patients included were required to have at least 1 prescription fill for prucalopride on or after April 2, 2019 (ie, the prucalopride launch date in the United States). The index date was the date of the first prescription fill for prucalopride. The baseline period was defined as the 6 months before the index date. The follow-up period was defined as the period from the index date to the end of continuous eligibility. Eligible patients had continuous health plan enrollment for at least 6 months before and at least 6 months after the index date. Patients were also required to have at least 1 constipation-related International Classification of Diseases, 10th Revision, Clinical Modification diagnosis code (Table A1) during the baseline or follow-up periods and were aged 18 years or older at index. The study design is shown in Figure A1. The exclusion criteria, assessed over the baseline and follow-up periods, included: at least 1 diagnosis code for irritable bowel syndrome with constipation, drug-induced constipation, postoperative ileus, or opioid supply for 45 days or more (Table A2). HCRU and direct health care costs were compared in eligible patients during the 6 months before (baseline period) and the 6 months after (study period) the initiation of prucalopride treatment.

### HCRU

HCRU was assessed in patients aged 18 years and older from the IBM MarketScan CCAE and MDCR Databases,^19^ and included:

• all-cause and constipation-related outpatient visits (including any, office and clinic or urgent care visits, hospital and surgical center visits, and specialist visits (for all-cause only))
• all-cause and constipation-related emergency room (ER) visits
• all-cause and constipation-related inpatient visits
• constipation-related pharmacy utilization (including secretagogues, laxatives, enemas, prokinetics (other than prucalopride), and antidepressants)
• plain film radiography.

The severity of patients’ constipation symptoms was not captured in the claims databases.

Constipation-related HCRU was defined as HCRU for which a diagnosis code for constipation was 1 of the first 3 codes selected for the corresponding claim. Constipation-related pharmacy utilization encompassed all pharmacy claims that had a National Drug Code or a Generic Product Identifier code corresponding to constipation-related treatments (Table A3). Plain film radiography was identified using Current Procedural Terminology codes (Table A4).

### Direct Health Care Costs

Patients aged 18–64 years from the MarketScan CCAE Database^19^ with no capitated health plan were included in the health care costs analysis. Patients aged over 64 years were excluded because data were not available on or after January 1, 2019, for these patients. Direct health care costs were estimated using a third-party payer perspective, adjusted to 2020 US dollars using the Consumer Price Index for medical care,^22^ and comprised:

• total all-cause health care costs (all-cause medical costs plus all-cause pharmacy costs)
  o total all-cause medical costs (including the costs associated with all-cause inpatient, ER, and outpatient visits)
  o total all-cause pharmacy costs
• total constipation-related health care costs (constipation-related medical costs plus constipation-related pharmacy costs)
  o total constipation-related medical costs (including the costs associated with constipation-related inpatient, ER, and outpatient visits)
  o total constipation-related pharmacy costs.

Health care costs in the outpatient setting included those for outpatient office and clinic or urgent care visits, outpatient hospital and surgical center visits, and other outpatient costs, such as those for laboratory services and outpatient home health services.

Constipation-related medical costs were defined as those associated with HCRU for which a constipation-related diagnosis code was 1 of the first 3 codes selected for the corresponding claim. Constipation-related pharmacy costs were defined as those associated with a National Drug Code or a Generic Product Identifier code corresponding to constipation-related treatments such as medications for CIC (any, lubiprostone, linaclotide, or plecanatide) and constipation-related medications (laxatives, enemas, or prokinetics (other than prucalopride)). Costs associated with OTC medications were not captured in the claims databases.

### Subgroup Analyses

Subgroup analyses were performed to assess the real-world HCRU and health care costs in patients with constipation who had used other prescription CIC medications (lubiprostone, linaclotide, or plecanatide) before the index date, and separately, in male patients with CIC.

### Statistical Analyses

Patient demographics and characteristics were summarized descriptively (N, percentage or mean, standard deviation (SD)). All-cause and constipation-related HCRU, pharmacy utilization, and plain film radiography reported as N (percentage) were compared during the baseline and study periods using McNemar’s tests, with statistical significance at 5%. Pharmacy utilization (number of distinct CIC medications used (other than prucalopride) and number of medication fills), number of plain film radiography tests (mean values only), number of all-cause and constipation-related visits, and direct health care costs were reported as mean (SD) and median (range). These continuous variables were compared during the baseline and study periods using the Wilcoxon signed-rank test, with statistical significance at 5%. Data analyses were conducted using R Version 3.6.3 and SAS Version 9.4 (SAS Institute, Inc, Cary, NC).

This study was based on deidentified Health Insurance Portability and Accountability Act–compliant data for which institutional review board review and formal consent were not required. No patient identifiable information was obtained during this study.

## Results

### Study Population

In total, 3134 patients with at least 1 prescription fill for prucalopride on or after April 2, 2019, were identified in the MarketScan CCAE and MDCR Databases^19^; of these, 690 were eligible for inclusion in the HCRU analysis (Figure A2A). Overall, 564 of 2910 patients identified in the MarketScan CCAE Database^19^ were eligible for inclusion in the health care costs analysis (Figure A2B). The majority of patients were female (HCRU analysis population, 87.5%; health care costs analysis population, 89.2%) (Table 1). The mean (SD) ages of patients from the HCRU and health care costs analysis populations were 48.0 (14.7) years and 45.6 (12.9) years, respectively (Table 1). Most patients from the HCRU and health care costs analysis populations were receiving prucalopride monotherapy (73.5% and 73.4%, respectively), as opposed to prucalopride in combination with another prescription therapy at the index date (Table 1). The proportion of patients who had not received a prescription for lubiprostone, linaclotide, or plecanatide before the index date was 36.8% in the HCRU analysis and 37.4% in the health care costs analysis. The most commonly reported comorbidities during the baseline period were gastroesophageal reflux disease (32.0%), hyperlipidemia (26.7%), anxiety (25.4%), hypertension (25.1%), depression (23.3%), and hypothyroidism (21.2%). Diagnoses of constipation reported during the baseline period included unspecified constipation (59.3%), other constipation (30.4%), CIC (18.8%), slow transit constipation (14.9%), and outlet dysfunction constipation (3.9%) (Table A5).

**Table 1:** Demographics and Characteristics of Patients Included in This Study at the Index Date

Demographic or characteristic	HCRU analysis population (aged ≥ 18 y) (N = 690)	Health care costs analysis population (aged 18–64 y) (N = 564)
Sex, n (%)		
Female	604 (87.5)	503 (89.2)
Male	86 (12.5)	61 (10.8)
Age, y, mean (SD)	48.0 (14.7)	45.6 (12.9)
Age category, y, n (%)		
18–34	129 (18.7)	115 (20.4)
35–44	122 (17.7)	110 (19.5)
45–54	188 (27.2)	164 (29.1)
55–64	195 (28.3)	175 (31.0)
≥ 65	56 (8.1)	0 (0.0)
US region, n (%)		
South	332 (48.1)	290 (51.4)
Northeast	198 (28.7)	147 (26.1)
North central	98 (14.2)	74 (13.1)
West	60 (8.7)	53 (9.4)
Unknown	2 (0.3)	0 (0.0)
Insurance plan type, n (%)		
Basic and major medical, comprehensive, or EPO	26 (3.8)	16 (2.8)
HMO	60 (8.7)	0 (0.0)
POS	54 (7.8)	54 (9.6)
PPO	408 (59.1)	358 (63.5)
CDHP	81 (11.7)	81 (14.4)
HDHP	56 (8.1)	55 (9.8)
Unknown	5 (0.7)	0 (0.0)
Initial dose of prucalopride, n (%)		
1 mg	76 (11.0)	59 (10.5)
2 mg	614 (89.0)	505 (89.5)
Any other constipation-related prescription treatment before index date,^a^ n (%)	436 (63.2)	353 (62.6)
Index drugs,^b^ n (%)		
Prucalopride monotherapy	507 (73.5)	414 (73.4)
Prucalopride combination therapy	183 (26.5)	150 (26.6)
Prucalopride and lubiprostone	26 (3.8)	18 (3.2)
Prucalopride and linaclotide	118 (17.1)	100 (17.7)
Prucalopride and plecanatide	28 (4.1)	22 (3.9)
Prucalopride and > 1 other constipation-related prescription treatment^c^	11 (1.6)	10 (1.8)

CDHP, consumer-directed health plan; EPO, exclusive provider organization; HDHP, high deductible health plan; HMO, health maintenance organization; POS, point of service plan; PPO, preferred provider organization.

a Includes use of linaclotide, plecanatide, or lubiprostone. Treatment use was evaluated based on all claims before the index date (not restricted to the 6 months baseline period).

b Includes other constipation-related prescription treatments taken concurrently with prucalopride.

c Includes use of linaclotide, plecanatide, or lubiprostone.

### HCRU

#### Outpatient, ER, and inpatient visits

Claims for all-cause outpatient visits overall were identified for 99.3% of patients during the baseline period and for 98.4% of patients during the study period (*P* = .211) (Figure A). The proportions of patients who had claims for all-cause outpatient office and clinic or urgent care visits were 99.3% and 98.3% before and after initiation of prucalopride (*P* = .146), respectively (Figure A). Compared with baseline, significantly fewer patients had claims for all-cause outpatient hospital and surgical center visits (22.2% vs 14.6%; *P* < .001) and gastroenterologist visits (72.5% vs 48.7%; *P* < .001) after the initiation of prucalopride (Figure A). The proportions of patients with claims for all-cause dietitian, psychiatrist, ER, and inpatient visits did not change significantly after initiating prucalopride (Figure A). The mean (SD) number of all-cause outpatient office and clinic or urgent care visits (14.71 [12.09] vs 13.18 [12.24]), outpatient hospital and surgical center visits (0.35 [1.16] vs 0.23 [1.02]), or gastroenterologist visits (1.61 [1.61] vs 0.93 [1.36]) was significantly lower after initiation of prucalopride compared with before (each *P* < .001; Table 2).

**Figure:**
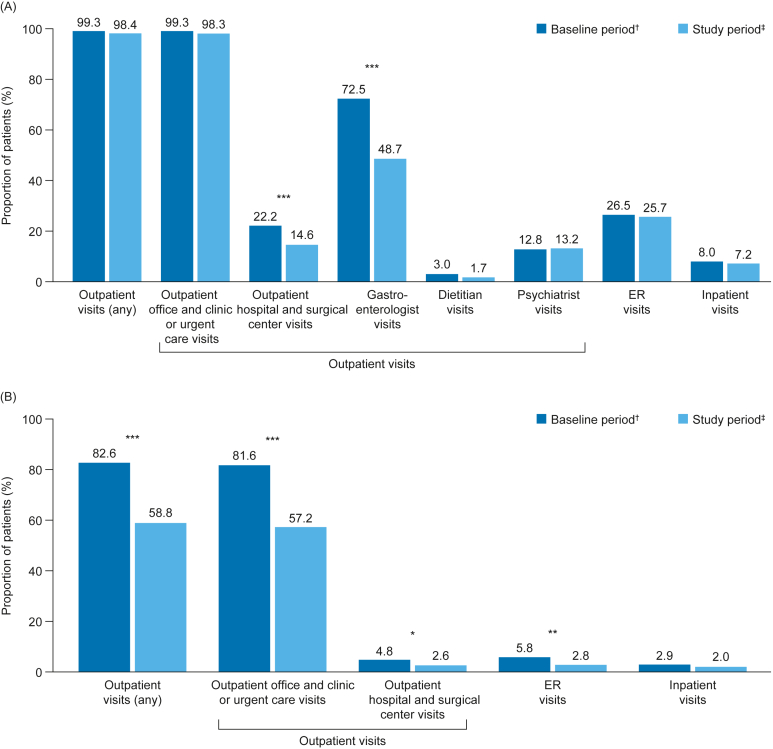
All-cause (A) and constipation-related (B) HCRU before and after prucalopride initiation in patients with CIC (N = 690). ^†^The baseline period was defined as the 6 months before the index date (the date of the first prescription fill for prucalopride). ^‡^The study period was defined as the 6 months after the index date (inclusive of the index date). ∗*P* < .05, ∗∗*P* < .01, ∗∗∗*P* < .001 (baseline period vs study period); *P* values were calculated using McNemar’s test (significance at 5%).

**Table 2:** Number of All-Cause and Constipation-Related Health Care Visits Before and After Prucalopride Initiation in Patients With CIC

Visits	Number of all-cause visits (N = 690)			Number of constipation-related visits (N = 690)		
	Mean (SD), median (range)			Mean (SD), median (range)		
	Baseline period^a^	Study period^b^	*P* value^c^	Baseline period^a^	Study period^b^	*P* value^c^
Outpatient	16.30 (15.07), 12.00 (0.00–161.00)	14.97 (15.42), 11.00 (0.00–157.00)	<.001	2.26 (6.13), 1.00 (0.00–149.00)	1.52 (5.04), 1.00 (0.00–116.00)	<.001
Outpatient office and clinic or urgent care	14.71 (12.09), 11.00 (0.00–109.00)	13.18 (12.24), 10.00 (0.00–119.00)	<.001	1.98 (2.45), 1.00 (0.00–30.00)	1.27 (2.45), 1.00 (0.00–33.00)	<.001
Outpatient hospital and surgical center	0.35 (1.16), 0.00 (0.00–16.00)	0.23 (1.02), 0.00 (0.00–20.00)	<.001	0.05 (0.21), 0.00 (0.00–1.00)	0.04 (0.29), 0.00 (0.00–6.00)	–
Gastroenterologist	1.61 (1.61), 1.00 (0.00–10.00)	0.93 (1.36), 0.00 (0.00–13.00)	<.001	–	–	–
Dietitian	0.10 (0.86), 0.00 (0.00–18.00)	0.06 (0.63), 0.00 (0.00–11.00)	–	–	–	–
Psychiatrist	0.47 (2.04), 0.00 (0.00–30.00)	0.48 (2.14), 0.00 (0.00–37.00)	.767	–	–	–
ER	0.53 (1.32), 0.00 (0.00–20.00)	0.50 (1.15), 0.00 (0.00–14.00)	.725	0.08 (0.35), 0.00 (0.00–4.00)	0.03 (0.20), 0.00 (0.00–2.00)	–
Inpatient	0.11 (0.41), 0.00 (0.00–3.00)	0.10 (0.41), 0.00 (0.00–6.00)	.631	0.03 (0.18), 0.00 (0.00–2.00)	0.02 (0.16), 0.00 (0.00–2.00)	–
Total hospitalization days	0.93 (6.33), 0.00 (0.00–97.00)	0.83 (6.93), 0.00 (0.00–160.00)	.711	0.21 (1.66), 0.00 (0.00–26.00)	0.45 (6.28), 0.00 (0.00–156.00)	–

a The baseline period was defined as the 6 months before the index date (the date of the first prescription fill for prucalopride).

b The study period was defined as the 6 months after the index date (inclusive of the index date).

c *P* values were calculated using the Wilcoxon signed-rank test (significance at 5%).

Claims for constipation-related outpatient visits overall were identified for 82.6% of patients in the baseline period and for 58.8% of patients during the study period (*P* < .001) (Figure B). Compared with baseline, the proportion of patients who had claims for constipation-related visits was significantly lower after initiation of prucalopride for outpatient office and clinic or urgent care visits (81.6% vs 57.2%; *P* < .001), outpatient hospital and surgical center visits (4.8% vs 2.6%; *P* < .05), and ER visits (5.8% vs 2.8%; *P* < .01) (Figure B). The proportion of patients who had constipation-related inpatient visits was also numerically reduced after initiation of prucalopride compared with baseline, although this did not meet statistical significance (Figure B). Compared with baseline, the number of constipation-related outpatient office and clinic or urgent care visits was significantly lower after initiation of prucalopride (*P* < .001; Table 2).

#### Pharmacy utilization

The mean (SD) number of CIC medications other than prucalopride that patients were receiving significantly decreased from 0.53 (0.65) during the baseline period to 0.26 (0.50) after prucalopride initiation (*P* < .001) (Table A6). There was a significant reduction from baseline in the proportion of patients who had prescription fills for laxatives after the initiation of prucalopride (24.1% vs 12.0%; *P* < .001) (Table A6). Further information on pharmacy utilization is provided in the Supplementary Material (Supplementary Results).

#### Plain film radiography

Compared with baseline, there was a significant reduction in the proportion of patients who had claims for plain film radiography after initiation of prucalopride (19.7% vs 8.8%; *P* < .001). Compared with baseline, the mean (SD) number of plain film radiography tests was also significantly reduced in the study period (0.36 [0.95] vs 0.17 [0.87]; *P* < .001).

### Direct Health Care Costs

#### All-cause medical costs

Compared with baseline, total all-cause health care costs were numerically lower after the initiation of prucalopride (mean: $21,145 vs $20,263; median: $6583 vs $6284) (Table 3). However, from baseline, there was a significant reduction in total all-cause medical costs after the initiation of prucalopride (mean: $13,393 vs $11,679; median: $3502 vs $2659; *P* < .001; Table 3). This decrease was primarily driven by a significant reduction in outpatient office and clinic or urgent care costs (mean: $5860 vs $5324; median: $2421 vs $1703; *P* < .001) and numerical reductions in inpatient costs (mean: $4921 vs $3819; median: $0 vs $0; *P* = .327) after the initiation of prucalopride (Table 3). The outpatient hospital and surgical center costs were also significantly reduced after the initiation of prucalopride (mean: $482 vs $315; median: $0 vs $0; *P* < .05; Table 3).

**Table 3:** All-Cause and Constipation-Related Direct Health Care Costs Before and After Prucalopride Initiation in Patients With CIC Who Did Not Have a Capitated Health Plan (N = 564)

Cost type	All-cause			Constipation-related		
	Mean (SD), median (range)			Mean (SD), median (range)		
	Baseline period^a^	Study period^b^	*P* value^c^	Baseline period^a^	Study period^b^	*P* value^c^
Total	21,145 (97,801), 6583 (0–1,883,580)	20,263 (77,854), 6284 (170–1,554,349)	.874	1497 (2732), 667 (0–26,410)	2332 (3077), 1906 (0–53,399)	<.001
Pharmacy	7752 (77,800), 1528 (0–1,839,590)	8584 (63,291), 2779 (0–1,488,141)	<.001	621 (926), 13 (0–6390)	1751 (1292), 1657 (0–8727)	<.001
Medical	13,393 (57,007), 3502 (0–1,266,786)	11,679 (40,481), 2659 (0–816,432)	<.001	876 (2550), 150 (0–25,793)	580 (2810), 41 (0–52,486)	<.001
Outpatient	7216 (12,698), 3029 (0–145,439)	6878 (15,038), 2092 (0–152,609)	<.001	550 (1376), 131 (0–20,695)	385 (1117), 36 (0–11,421)	<.001
Outpatient other^d^	875 (4517), 43 (0–71,794)	1239 (6292), 25 (0–61,505)	.057	57 (903), 0 (0–20,695)	30 (487), 0 (0–11,421)	.432
Outpatient hospital and surgical center	482 (1684), 0 (0–25,668)	315 (1088), 0 (0–12,049)	<.05	55 (287), 0 (0–2414)	39 (317), 0 (0–5140)	.135
Outpatient office and clinic or urgent care	5860 (10,841), 2421 (0–145,439)	5324 (12,051), 1703 (0–152,609)	<.001	439 (936), 124 (0–6743)	316 (946), 24 (0–8840)	<.001
Inpatient	4921 (52,534), 0 (0–1,213,240)	3819 (36,172), 0 (0–810,621)	.327	170 (1801), 0 (0–25,793)	156 (2327), 0 (0–45,825)	.577
ER	1256 (3827), 0 (0–45,076)	982 (3016), 0 (0–26,987)	.077	156 (920), 0 (0–11,909)	39 (317), 0 (0–4687)	<.01

All costs are reported in US dollars.

Numbers may not sum to the total costs, owing to rounding and gamma distribution of the data.

a The baseline period was defined as the 6 months before the index date (the date of the first prescription fill for prucalopride).

b The study period was defined as the 6 months after the index date (inclusive of the index date).

c *P* values were calculated using the Wilcoxon signed-rank test (significance at 5%).

d Other outpatient costs included laboratory tests and outpatient home health services.

#### All-cause pharmacy costs

Compared with baseline, all-cause pharmacy costs (including all treatment-related costs) significantly increased after the initiation of prucalopride (mean: $7752 vs $8584; median: $1528 vs $2779; *P* < .001; Table 3).

### Constipation-Related Health Care Costs

#### Constipation-related medical costs

Compared with baseline, total constipation-related costs significantly increased after the initiation of prucalopride (mean: $1497 vs $2332; median: $667 vs $1906; *P* < .001; Table 3); however, when the cost of prucalopride was excluded from the calculation, these total constipation-related costs were significantly reduced (mean: $1497 vs $949; median: $667 vs $117; *P* < .001). From baseline, the total constipation-related medical costs were also significantly reduced after the initiation of prucalopride (mean: $876 vs $580; median: $150 vs $41; *P* < .001; Table 3). This reduction can be mostly attributed to the significant decrease in constipation-related outpatient office and clinic or urgent care costs (mean: $439 vs $316; median: $124 vs $24; *P* < .001; Table 3).

#### Constipation-related pharmacy costs

Compared with baseline, constipation-related pharmacy costs after the initiation of prucalopride significantly increased overall (mean: $621 vs $1751; median: $13 vs $1657; *P* < .001; Table 3); however, when the cost of prucalopride was excluded from the calculation, constipation-related pharmacy costs were significantly reduced from baseline after the initiation of prucalopride (mean: $621 vs $369; median: $13 vs $0; *P* < .001).

### Analysis of HCRU and Direct Health Care Costs in the Subpopulations of Interest

#### Patients with CIC who had used other CIC medications before the index date

Of 690 patients, 63.2% (N = 436) and 51.2% (N = 353) were included in the HCRU and health care costs analyses, respectively, for the subgroup who had used other CIC medications before the index date. The mean (SD) number of CIC medications other than prucalopride that patients were receiving significantly decreased after prucalopride initiation (0.84 [0.64] vs 0.36 [0.58]; *P* < .001). Compared with baseline, significant reductions were observed in the proportion of patients with all-cause outpatient hospital and surgical center visits and gastroenterologist visits (*P* < .05 and *P* < .001, respectively; Figure A3A) and constipation-related outpatient office and clinic or urgent care visits after prucalopride initiation (*P* < .001; Figure A3B). From baseline, the number of all-cause and constipation-related outpatient visits was significantly reduced after the initiation of prucalopride, which was primarily driven by reductions in outpatient office and clinic or urgent care visits (Table A7).

Total all-cause medical costs significantly increased after the initiation of prucalopride (mean: $11,547 vs $12,055; median: $3913 vs $2880; *P* < .01), although significant reductions in the outpatient office and clinic or urgent care costs were observed (*P* < .001; Table A8). Total constipation-related medical costs were significantly reduced after the initiation of prucalopride (mean: $976 vs $654; median: $174 vs $51; *P* < .001), with significant reductions in the outpatient office and clinic or urgent care and ER costs observed (Table A8). Constipation-related pharmacy costs increased after the initiation of prucalopride (mean: $982 vs $2010; median: $817 vs $1906; *P* < .001; Table A8). However, when the cost of prucalopride was excluded from the calculation, constipation-related pharmacy costs significantly reduced from baseline after the initiation of prucalopride (mean: $982 vs $526; median: $817 vs $0; *P* < .001).

#### Male patients with CIC

Of 690 patients, 12.5% (N = 86) and 8.8% (N = 61) were included in the HCRU and health care costs analyses, respectively, for the male subgroup. Compared with baseline, the proportion of patients with all-cause HCRU associated with gastroenterologist visits and constipation-related HCRU associated with an outpatient office and clinic or urgent care visit were significantly reduced during the 6 months after prucalopride initiation (*P* < .01 and *P* < .05, respectively); no other statistically significant differences were observed (Figure A4A and B). From baseline, the number of all-cause gastroenterologist visits and constipation-related outpatient office and clinic or urgent care visits were significantly reduced during the 6 months after prucalopride initiation (*P* < .01 and *P* < .05, respectively; Table A9).

Compared with baseline, total all-cause and constipation-related medical costs were significantly reduced during the 6 months after prucalopride initiation (all-cause: mean, $18,252 vs $12,157; median, $3287 vs $2345; *P* < .05; constipation-related: mean, $1723 vs $513; median: $230 vs $6; *P* < .05; Table A10). Significant reductions in all-cause outpatient medical costs were also observed (mean: $8753 vs $7389; median: $2544 vs $1943; *P* < .05; Table A10). Constipation-related pharmacy costs were significantly increased during the 6 months after prucalopride initiation (mean: $468 vs $1606; median: $0 vs $1305; *P* < .001; Table A10). However, when the cost of prucalopride was excluded from the calculation, constipation-related pharmacy costs were significantly reduced from baseline after the initiation of prucalopride (mean: $468 vs $296; median: $0 vs $0; *P* < .05).

## Discussion

The economic burden associated with constipation-related health care is substantial, with an estimated annual direct health care expenditure of greater than $230 million in the United States in 2001.^23^^,^^24^ Current costs associated with constipation-related HCRU are expected to be even higher. This observational cohort study evaluated the association between prucalopride initiation and real-world all-cause and constipation-related HCRU and direct health care costs 6 months after treatment initiation for CIC in adults in the United States. After prucalopride initiation, lower all-cause and constipation-related HCRU was observed in patients compared with baseline. For all-cause HCRU, the proportion of patients who had claims for outpatient hospital and surgical center visits and gastroenterologist visits significantly reduced after prucalopride initiation. For constipation-related HCRU, the proportion of patients who had claims for outpatient visits (both office and clinic or urgent care visits and hospital and surgical center visits) and ER visits significantly reduced following prucalopride initiation. As anticipated, total constipation-related costs and constipation-related pharmacy costs were increased during the study period compared with the baseline period owing to the initiation of prucalopride. The additional costs associated with prucalopride may be offset by the treatment benefits in the long term, particularly for total constipation-related costs. Despite the increases in pharmacy costs observed, all-cause and constipation-related medical costs were significantly reduced after prucalopride initiation, primarily driven by reductions in outpatient office and clinic or urgent care costs.

The HCRU and health care costs associated with CIC in the United States are poorly characterized. A 2020 systematic literature review identified only 3 studies describing the HCRU and economic burden related to CIC.^25^ HCRU and health care costs are substantially elevated in patients with constipation compared with individuals without constipation,^5^^,^^7^ with patients incurring an additional direct health care cost of $3508 (2010 US dollars) per year due to their condition compared with healthy controls.^6^ Cai et al. (2014) observed that costs related to medical services, including inpatient admissions, ER visits, physician office visits, and other outpatient services, accounted for almost 80% of the mean annual all-cause costs ($9012 per year [2010 $]) for patients with chronic constipation.^6^ However, only one other study appears to have examined the real-world impact of a prescription medication on the HCRU and economic burden in CIC or other gastrointestinal-related conditions and reported similar findings to ours.^26^ Taylor et al. (2018) noted an increase in pharmacy costs, but substantial reductions in HCRU and health care costs associated with gastrointestinal-related outpatient visits (including CIC and irritable bowel syndrome) following the initiation of linaclotide.^26^

In addition, we examined the association of prucalopride initiation with HCRU and health care costs in patients with CIC who had previously used other CIC medications. In this subpopulation, HCRU and health care costs associated with constipation-related outpatient office and clinic or urgent care visits and costs associated with constipation-related ER visits were reduced significantly during the 6 months after prucalopride initiation. It is unclear from our study why these patients initiated prucalopride. One possible explanation is that they may have initiated a new treatment owing to refractory symptoms while receiving previous CIC medications; further investigations are warranted to confirm this hypothesis. Our analyses did not differentiate between patients who had received only 1 prior CIC medication versus patients who had received more than 1, and therefore our data cannot provide potential insights into responsiveness to therapy. Furthermore, most patients in our study were female, which is expected, given that CIC is commonly observed in women.^27^ Therefore, we investigated the economic burden incurred by the initiation of a new treatment in male patients, a patient demographic that is often overlooked in the literature. At baseline, all male patients had used an outpatient service for any cause, with ∼80% of patients attending any outpatient services for reasons related to constipation. In fact, at baseline, mean all-cause and constipation-related medical costs for this subpopulation were numerically higher than the costs reported for the total population; however, statistical testing was not performed.

Our findings provide the first real-world data on the association of prucalopride initiation with HCRU and direct health care costs in adults with CIC, with data collected from 2 high-quality and robust nationally representative databases.

### Limitations

Study limitations include those inherent to analyses of claims data, such as the inability to capture information on disease severity, OTC medication use and the associated costs, and a potential lack of generalizability of data from commercially insured populations to other patient populations. In addition, only data pertaining to plain film radiography were included as part of the HCRU analysis, given that clinicians increasingly rely on radiographs to assess stool burden in patients with constipation.^28^ While routine diagnostic testing is not generally recommended for the evaluation of chronic constipation,^29^^,^^30^ patients are often referred for colonoscopy as part of defensive clinical practices.^31^ Patients were required to have at least 1 prescription fill; however, our analyses did not differentiate between patients with only a single prescription fill and those with many prescription fills. As part of the study, data on medication persistence and adherence have been published elsewhere.^32^ Potential confounding factors such as seasonality, disease progression and severity, and concurrent use of other medications were not controlled for in this study, limiting generalizability to other populations and settings. Another limitation was the potential for coding inaccuracies and omissions when diagnosis and procedure codes were reported for administrative purposes. Finally, owing to the IBM MarketScan Database being unable to fully capture the Medicare costs for patients aged 65 years or older, health care costs for this age population were not reported. Further studies with longer follow-up periods in this patient population should therefore be conducted.

## Conclusion

The proportions of patients who had all-cause HCRU associated with outpatient hospital and surgical center and gastroenterologist visits, and constipation-related HCRU associated with any outpatient and ER visits significantly decreased after prucalopride initiation compared with before. Total constipation-related health care costs increased following prucalopride initiation, which was primarily driven by pharmacy costs, as would be anticipated when starting a new treatment; however, despite the increase in pharmacy costs, all-cause and constipation-related medical costs were significantly reduced. Additional real-world studies are required to confirm these findings with prucalopride over a period longer than 6 months.

## Supplementary Materials

Figure A1

Figure A2

Figure A3

Figure A4
