# Supplementary figures and images for: Economic Burden of Patients With Chronic Idiopathic Constipation in the USA Before and After Prucalopride Initiation
Source: Gastro Hep Adv. 2025 Mar 24;4(7):100664. doi: 10.1016/j.gastha.2025.100664 (PMC12148438; doi:10.1016/j.gastha.2025.100664)

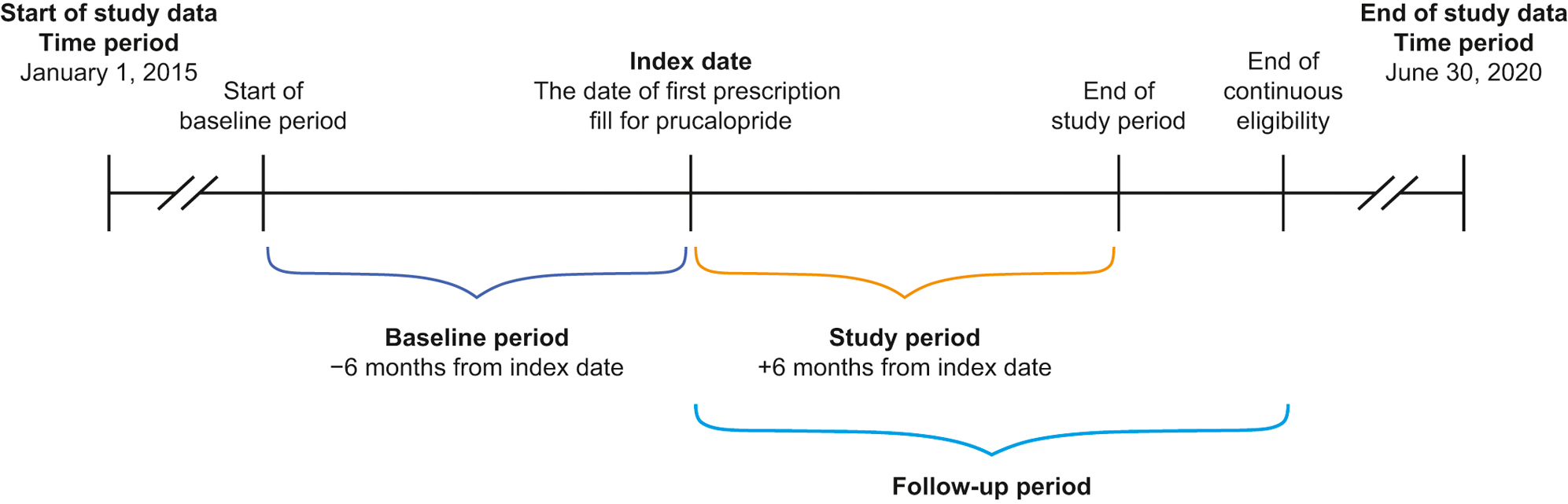

Supplement: Figure A1 [file figs1.jpg]

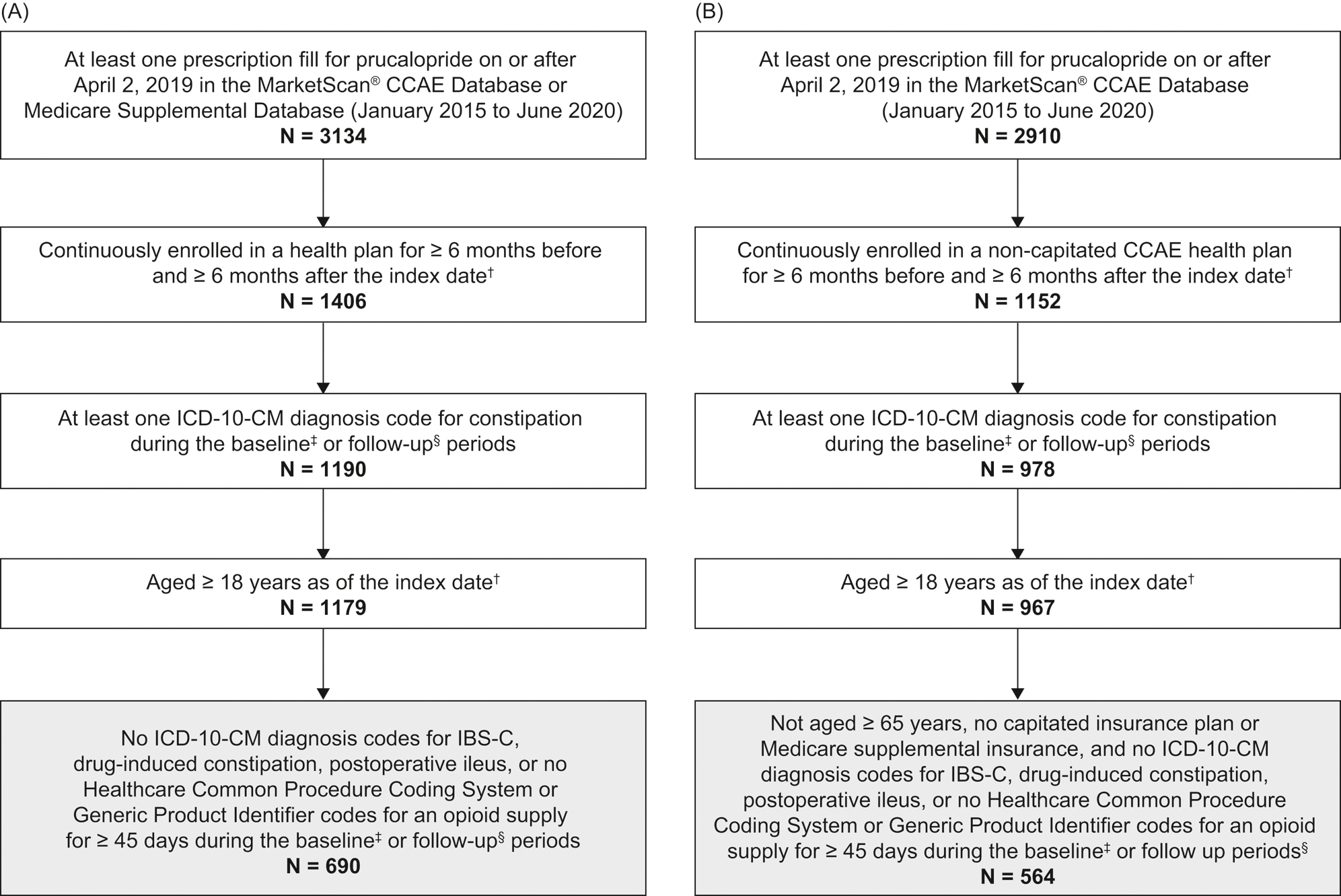

Supplement: Figure A2 [file figs2.jpg]

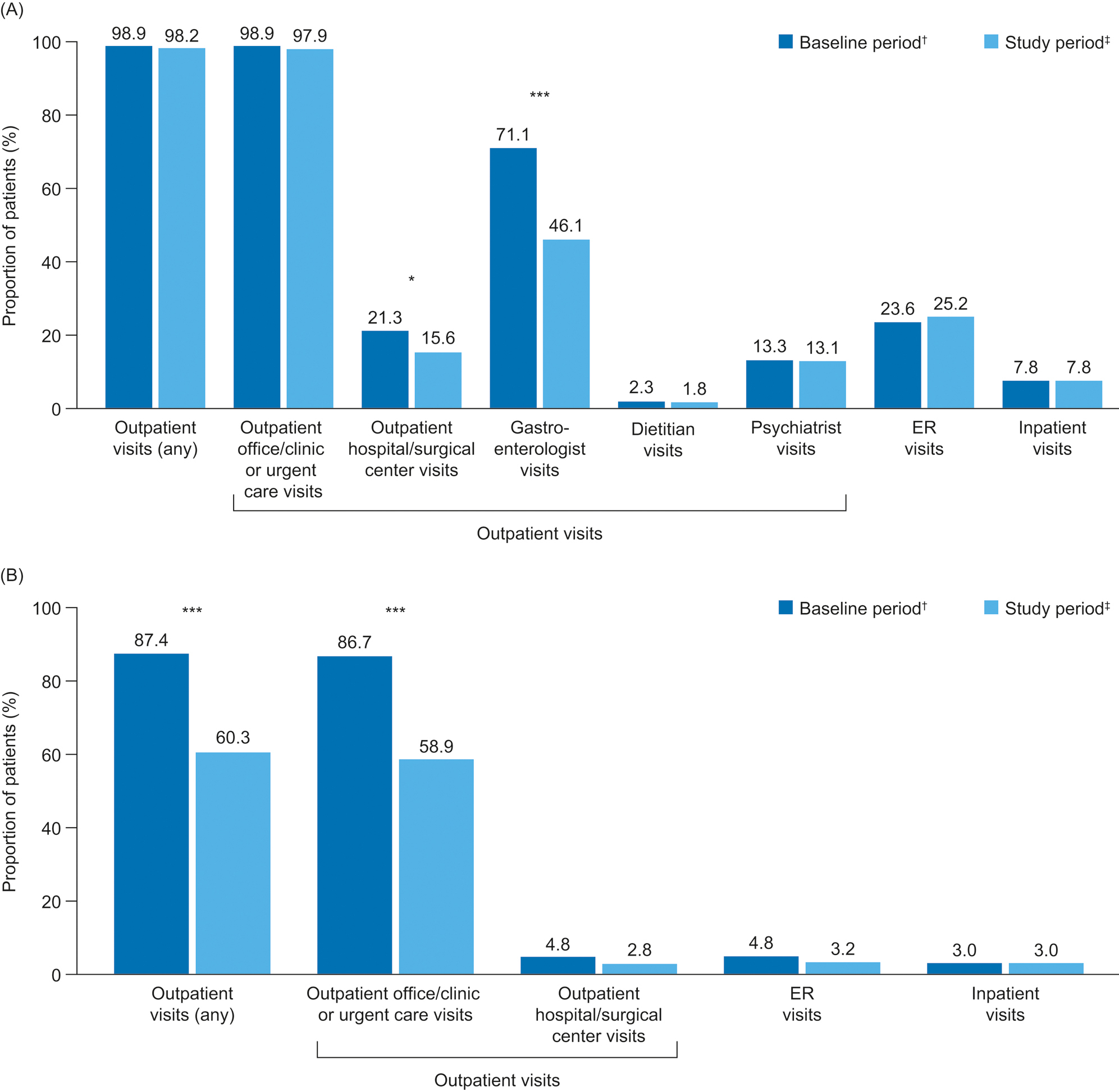

Supplement: Figure A3 [file figs3.jpg]

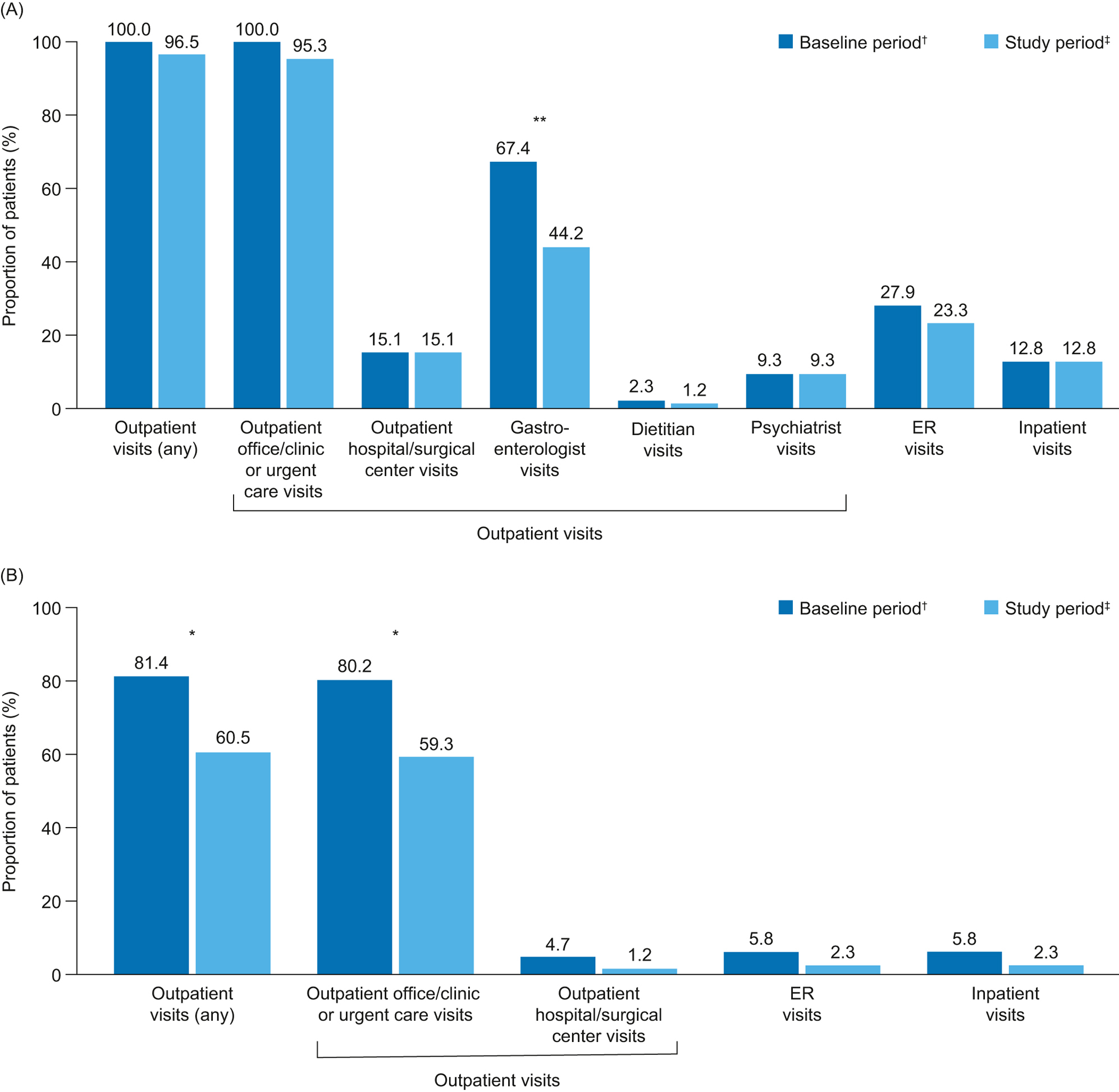

Supplement: Figure A4 [file figs4.jpg]
