# Supplementary material for: Economic Burden of Patients With Chronic Idiopathic Constipation in the USA Before and After Prucalopride Initiation
Source: Gastro Hep Adv. 2025 Mar 24;4(7):100664. doi: 10.1016/j.gastha.2025.100664 (PMC12148438; doi:10.1016/j.gastha.2025.100664)
Supplement: Supplementary material [file mmc1.docx]

**Supplementary material**

**Economic Burden of Patients with Chronic Idiopathic Constipation in the USA Before and After Prucalopride Initiation**

**Short running title:** Effect of prucalopride on HCRU and costs

Paul Feuerstadt,^1,2^ Mei Lu,^3^ Emi Terasawa,^4^ Brian Terreri,^3^ Shawn Du,^4^ Selina Pi,^4^ Ben Westermeyer,^4^ Rajeev Ayyagari,^5^ Anthony Lembo,^6^ Baharak Moshiree,^7^ Mena Boules,^3^ and Brooks D. Cash^8^

^1^Physicians Alliance of Connecticut Gastroenterology Center, Hamden, Connecticut

^2^Yale Division of Digestive Diseases, Yale School of Medicine, Yale University, New Haven, Connecticut

^3^Takeda Pharmaceuticals USA, Inc., Lexington, Massachusetts

^4^Analysis Group, Inc., New York, New York

^5^Analysis Group, Inc., Boston, Massachusetts

^6^Digestive Disease & Surgery Institute, Cleveland Clinic, Cleveland, Ohio

^7^Department of Medicine, Atrium Health Wake Forest University School of Medicine, Charlotte, North Carolina

^8^Division of Gastroenterology, Hepatology and Nutrition, University of Texas Health Science Center at Houston, Houston, Texas

**Supplementary Results**

**Pharmacy Utilization**

**Supplementary Figures and Tables**

**Figure A1.** Study design.

**Figure A2.** Selection algorithm for patients with CIC who were receiving prucalopride for the healthcare resource utilization **(A)** and direct healthcare costs **(B)** analyses.

**Figure A3.** All-cause **(A)** and constipation-related **(B)** HCRU before and after prucalopride initiation in patients with CIC who previously used other CIC medications (N = 436).

**Figure A4.** All-cause **(A)** and constipation-related **(B)** HCRU before and after prucalopride initiation in male patients with CIC (N = 86).

**Table A1.** Constipation-related Diagnosis Codes for Inclusion Criteria and Constipation-related HCRU

**Table A2.** Diagnosis, Procedure, and Drug Codes for Exclusion Criteria

**Table A3.** Drug Codes for Treatments of Interest

**Table A4.** Procedure Codes for Plain Film Radiography

**Table A5.** Baseline Period Comorbidities of Patients with CIC Included in This Study (N = 690)

**Table A6.** Pharmacy Utilization Before and After Prucalopride Initiation in Patients with CIC (N = 690)

**Table A7.** Number of All-cause and Constipation-related Health-care Visits Before and After Prucalopride Initiation in Patients with CIC Who Previously Used Other CIC Medications

**Table A8.** All-cause and Constipation-related Direct Health-care Costs Before and After Prucalopride Initiation in Patients with CIC Who Did Not Have a Capitated Health Plan and Who Previously Used Other CIC Medications (N = 353)

**Table A9.** Number of All-cause and Constipation-related Health-care Visits Before and After Prucalopride Initiation in Male Patients with CIC

**Table A10.** All-cause and Constipation-related Direct Health-care Costs Before and After Prucalopride Initiation in Male Patients With CIC Who Did Not Have a Capitated Health Plan (N = 61)

***Supplementary Results***

**Pharmacy utilization**

Compared with baseline, the proportion of patients who reported secretagogue (lubiprostone, linaclotide, or plecanatide) prescription fills was significantly lower after initiation of prucalopride (45.1% vs 22.6%; *P* < .001; Table A6). The proportion of patients who had claims for prokinetics (other than prucalopride) was 7.5% before and 5.4% after prucalopride initiation (*P* = .054). Prescription fills for antidepressants were similar during the baseline and study periods (42.8% and 44.8%, respectively; *P*= .151; Table A6).

***Supplementary Figures and Tables***

**Figure A1.** Study design.


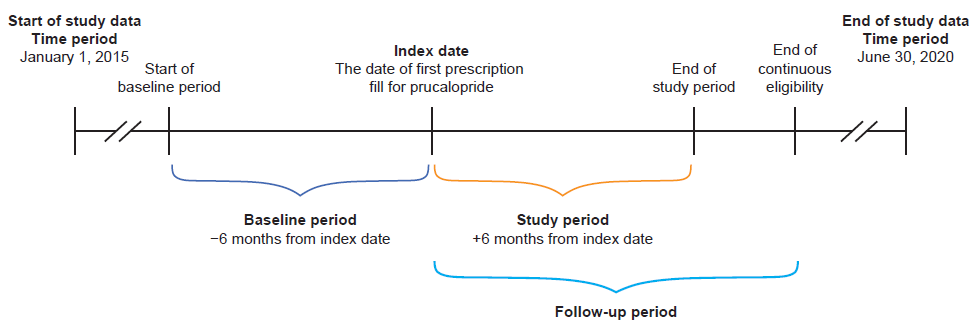


Prucalopride was approved by the Food and Drug Administration on December 17, 2018, and was made commercially available in the United States on April 2, 2019. Therefore, the earliest possible start date for the baseline period was October 1, 2018.

**Figure A2.** Selection algorithm for patients with CIC who were receiving prucalopride for the health-care resource utilization **(A)** and direct health-care costs **(B)** analyses.


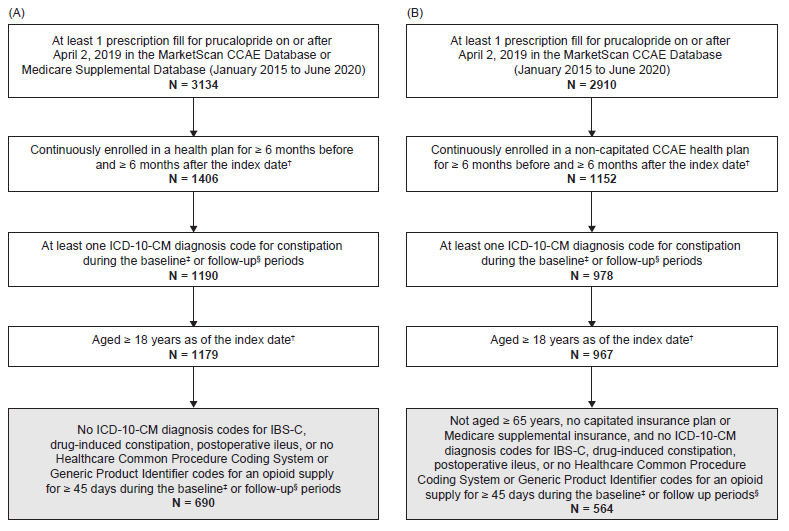


^†^The index date was defined as the date of the first prescription fill for prucalopride.

^‡^The baseline period was defined as the 6 months before the index date.

^§^The follow-up period was defined as the period from the index date to the end of continuous eligibility.

CCAE, Commercial Claims and Encounters; CIC, chronic idiopathic constipation; IBS-C, irritable bowel syndrome with constipation; ICD-10-CM, International Classification of Diseases, Tenth Revision, Clinical Modification.

**Figure A3.** All-cause **(A)** and constipation-related **(B)** HCRU before and after prucalopride initiation in patients with CIC who previously used other CIC medications (N = 436).


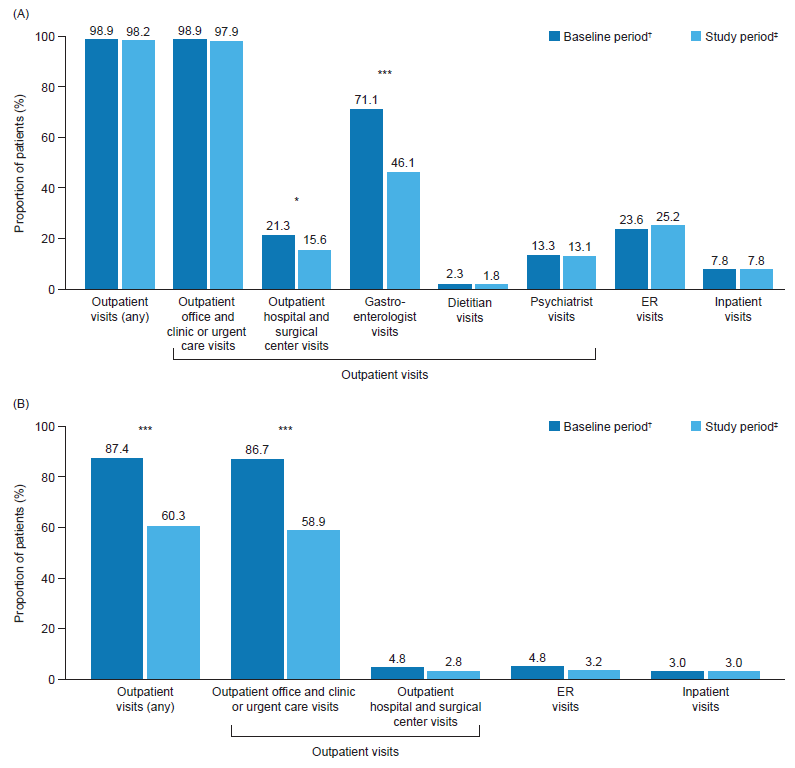


^†^The baseline period was defined as the 6 months before the index date (the date of the first prescription fill for prucalopride).

^‡^The study period was defined as the 6 months after the index date (inclusive of the index date).

**P* < .05, ****P* < .001 (baseline period vs study period); *P* values were calculated using McNemar’s test (significance at 5%).

CIC, chronic idiopathic constipation; ER, emergency room; HCRU, health-care resource utilization.

**Figure A4.** All-cause **(A)** and constipation-related **(B)** HCRU before and after prucalopride initiation in male patients with CIC (N = 86).


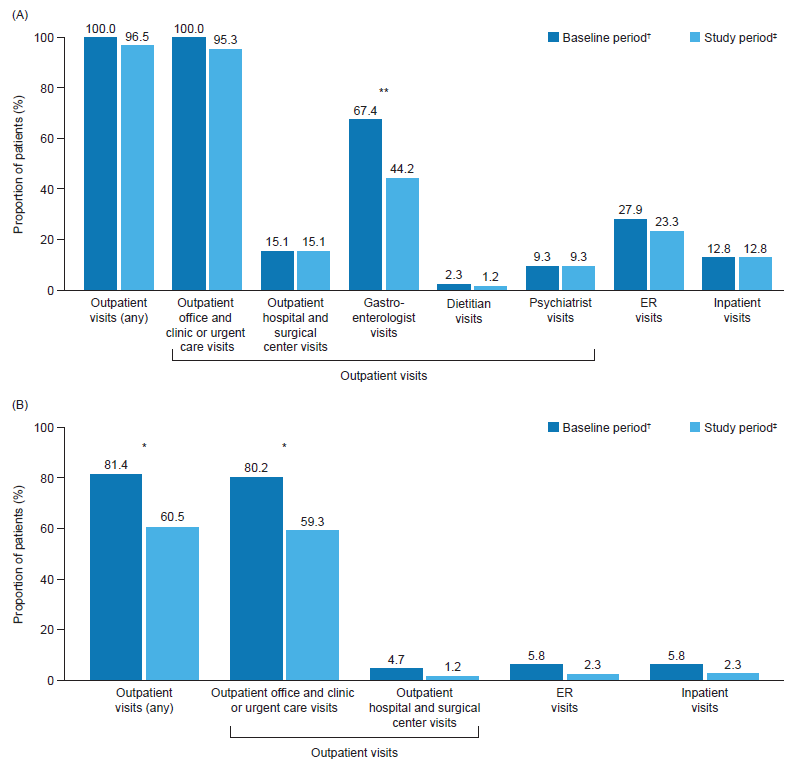


^†^The baseline period was defined as the 6 months before the index date (the date of the first prescription fill for prucalopride).

^‡^The study period was defined as the 6 months after the index date (inclusive of the index date).

**P*< .05; ***P* < .01, (baseline period vs study period); *P* values were calculated using McNemar’s test (significance at 5%).

CIC, chronic idiopathic constipation; ER, emergency room; HCRU, health-care resource utilization.

**Table A1.** Constipation-related Diagnosis Codes for Inclusion Criteria and Constipation-related HCRU

| **Constipation diagnosis** | **ICD-10-CM** |
| --- | --- |
| Constipation, unspecified | K59.00 |
| Slow transit constipation | K59.01 |
| Outlet dysfunction constipation | K59.02 |
| Chronic idiopathic constipation | K59.04 |
| Other constipation | K59.09 |

HCRU, health-care resource utilization; ICD-10-CM, International Classification of Diseases, Tenth Revision, Clinical Modification.

**Table A2.** Diagnosis, Procedure, and Drug Codes for Exclusion Criteria

| **Diagnosis, procedure, and drug use** | **ICD-10-CM** | **HCPCS** | **GPI** |
| --- | --- | --- | --- |
| Irritable bowel syndrome with constipation | K58.1 | – | – |
| Mixed irritable bowel syndrome | K58.2 | – | – |
| Irritable bowel syndrome without diarrhea | K58.9 | – | – |
| Drug-induced constipation | K59.03 | – | – |
| Opioids | – | J0745, J2270, J2271, J2275, S0093, J3010, J1170, S0092, J1960, J2175, J2180, J1230, S0109, J2410, J0592, J0595, S0009, S0012, J2300, J3070, J0570, J0571, J0572, J0573, J0574, J0575, Q9991, Q9992, Q0167, Q0168 | 65x |
| Post-operative ileus | K91.3x | – | – |

GPI, Generic Product Identifier; HCPCS, Healthcare Common Procedure Coding System; ICD‑10-CM, International Classification of Diseases, Tenth Revision, Clinical Modification.

**Table A3.** Drug Codes for Treatments of Interest

| **Treatment** | **NDC** | **GPI** |
| --- | --- | --- |
| **Prokinetics** | – | 5230x, 525550x, 5256x, 50100020x |
| Prucalopride | 54092054601, 54092054701, 54092054702, 54092054703 | – |
| **Secretagogues** |  |  |
| Lubiprostone | 00254302802, 00254302902, 16590047128, 16590047130, 16590047160, 16590047172, 16590047190, 16590062230, 16590062260, 16590062262, 16590062290, 35356050060, 47463010660, 54868597100, 54868615300, 55048010660, 63629459801, 63629639801, 64764008060, 64764024010, 64764024060, 68151500306 | – |
| Linaclotide | 00456120104, 00456120130, 00456120204, 00456120230, 00456120304, 00456120330, 55700040930 | – |
| Plecanatide | 65649000301, 65649000303, 65649000307, 65649000330, 70194000330, 70194010307 | – |
| **Laxatives** | – | 46x |
| Stimulant laxatives | – | 4620x |
| Pre-radiograph evacuation agents | – | 4620006010x, 4620006020x |
| Stool softeners | – | 4650x, 469910x |
| Suppositories |  |  |
| Glycerin | – | 466000100052x |
| Bisacodyl | – | 46200010005203, 46200010005205, 46200010006450, 46991002035220 |
| **Enemas** | – | 46109902105100, 46109902105110, 46200010005110, 46200010006405, 46200010006410, 46200010105100, 46400010005110, 46500010050105, 46500010055120, 46500010305103, 46500010305106, 46500010305110, 46600010005100, 46600010005120, 46600015005100, 46600015006400, 52500030005105, 52500030005110, 52500030206420, 89150010005110, 89150020105105, 89305010005100, 94401010105109, 94401010105110, 94401010105130, 94401010105150, 94401010105170, 94401010106484, 94401010106490 |
| **Antidepressants** | – | 58x |
| SSRIs | – | 5816x, 589985x |
| SNRIs | – | 5818x |
| Tricyclics | – | 5820x, 589960x, 589987x |
| MAOIs | – | 5810x |
| Phenylpiperazine | – | 5812x, 589980x |
| Tetracyclics | – | 5803x |
| Miscellaneous | – | 5830x, 589990x |

GPI, Generic Product Identifier; MAOI, monoamine oxidase inhibitor; NDC, National Drug Code; SNRI, serotonin and norepinephrine reuptake inhibitor; SSRI, selective serotonin reuptake inhibitor.

**Table A4.** Procedure Codes for Plain Film Radiography

|  | **CPT** |
| --- | --- |
| Plain film radiography | 74000, 74010, 74018, 74019, 74020, 74021, 74022 |

CPT, Current Procedural Terminology.

**Table A5.** Comorbidities Reported at Baseline for Patients with CIC Included in This Study (N = 690)

| **Comorbidities** | **Baseline period^a^**  **(N = 690)** |
| --- | --- |
| **CCI score, mean (SD)^b^** | 0.51 (1.03) |
| **Comorbidities used to calculate CCI scores^b^** |  |
| Chronic pulmonary disease | 85 (12.3) |
| Diabetes | 85 (12.3) |
| Rheumatic disease | 36 (5.2) |
| Mild liver disease | 36 (5.2) |
| Peripheral vascular disease | 27 (3.9) |
| Any malignancy^c^ | 25 (3.6) |
| Cerebrovascular disease | 22 (3.2) |
| Congestive heart failure | 17 (2.5) |
| Peptic ulcer disease | 13 (1.9) |
| Renal disease | 12 (1.7) |
| Hemiplegia or paraplegia | 8 (1.2) |
| Myocardial infarction | 5 (0.7) |
| Dementia | 2 (0.3) |
| Metastatic solid tumor | 2 (0.3) |
| Moderate to severe liver disease | 1 (0.1) |
| **Gastrointestinal comorbidities** |  |
| Gastroesophageal reflux disease | 221 (32.0) |
| Functional dyspepsia | 30 (4.3) |
| Small intestinal bacterial overgrowth | 12 (1.7) |
| Anal/rectal prolapse | 7 (1.0) |
| Crohn’s disease | 6 (0.9) |
| Gastrointestinal cancer | 6 (0.9) |
| Ulcerative colitis | 4 (0.6) |
| **Endocrine comorbidities** |  |
| Hypothyroidism | 146 (21.2) |
| Overweight or obesity | 83 (12.0) |
| **Psychiatric comorbidities** |  |
| Anxiety | 175 (25.4) |
| Depression | 161 (23.3) |
| **Other relevant comorbidities** |  |
| Hyperlipidemia | 184 (26.7) |
| Hypertension | 173 (25.1) |
| Fatigue | 127 (18.4) |
| Migraine | 91 (13.2) |
| Multiple sclerosis | 12 (1.7) |
| Ehlers-Danlos syndrome | 10 (1.4) |
| Stroke | 9 (1.3) |
| Parkinson’s disease | 5 (0.7) |
| Scleroderma | 5 (0.7) |
| Spinal cord injury | 1 (0.1) |
| **Constipation diagnoses** |  |
| Unspecified constipation | 409 (59.3) |
| Other constipation | 210 (30.4) |
| CIC | 130 (18.8) |
| Slow-transit constipation | 103 (14.9) |
| Outlet dysfunction constipation | 27 (3.9) |

Data are reported as n (%) unless indicated otherwise.

^a^The baseline period was defined as the 6 months before the index date (the date of the first prescription fill for prucalopride).

^b^The CCI was defined based on criteria by Charlson *et al.* and adapted by Quan *et al.^1,2^*

^c^Including lymphoma and leukemia, but excluding malignant neoplasm of skin.

CCI, Charlson comorbidity index; CIC, chronic idiopathic constipation; SD, standard deviation.

**Table A6.** Pharmacy Utilization Before and After Prucalopride Initiation in Patients with CIC (N = 690)

| **Pharmacy utilization** | **Baseline period**^a^  **(N = 690)** | **Study period**^b^  **(N = 690)** | ***P* value^c^** |
| --- | --- | --- | --- |
| **Number of distinct CIC medications used (other than prucalopride),**  **mean (SD), median (range)** | 0.53 (0.65),  0.00 (0.00–3.00) | 0.26 (0.50),  0.00 (0.00–3.00) | < .001 |
| **Any CIC medication (secretagogues), n (%)** | 311 (45.1) | 156 (22.6) | < .001 |
| Lubiprostone | 69 (10.0) | 26 (3.8) | < .001 |
| Linaclotide | 212 (30.7) | 113 (16.4) | < .001 |
| Plecanatide | 84 (12.2) | 37 (5.4) | < .001 |
| **Number of CIC medication fills, mean (SD), median (range)** | | | |
| Prucalopride | 0.00 (0.00)  0.00 (0.00–0.00) | 2.94 (1.93)  2.00 (1.00–11.00) | – |
| CIC medications (secretagogues) | 1.22 (1.81)  0.00 (0.00–10.00) | 0.61 (1.37)  0.00 (0.00–8.00) | < .001 |
| Lubiprostone | 0.22 (0.81)  0.00 (0.00–7.00) | 0.08 (0.45)  0.00 (0.00–4.00) | < .001 |
| Linaclotide | 0.73 (1.42)  0.00 (0.00–7.00) | 0.41 (1.14)  0.00 (0.00–8.00) | < .001 |
| Plecanatide | 0.26 (0.87)  0.00 (0.00–7.00) | 0.12 (0.60)  0.00 (0.00–6.00) | < .001 |
| **Constipation-related prescription fills, n (%)** | | | |
| Laxatives | 166 (24.1) | 83 (12.0) | < .001 |
| Bisacodyl suppositories | 0 (0.0) | 0 (0.0) | – |
| Glycerin suppositories | 0 (0.0) | 0 (0.0) | – |
| Stimulant laxatives | 6 (0.9) | 2 (0.3) | .221 |
| Pre-radiograph evacuation agents | 0 (0.0) | 0 (0.0) | – |
| Stool softeners | 3 (0.4) | 0 (0.0) | .248 |
| Other bowel evacuant agents^d^ | 137 (19.9) | 68 (9.9) | < .001 |
| Other laxatives (eg, other osmotic laxatives) | 39 (5.7) | 21 (3.0) | < .01 |
| Enemas | 0 (0.0) | 0 (0.0) | – |
| Prokinetics (other than prucalopride) | 52 (7.5) | 37 (5.4) | .054 |
| **Antidepressant prescription fills, n (%)** | | | |
| Antidepressants | 295 (42.8) | 309 (44.8) | .151 |
| SSRIs | 117 (17.0) | 124 (18.0) | .401 |

^a^The baseline period was defined as the 6 months before the index date (the date of the first prescription fill for prucalopride).

^b^The study period was defined as the 6 months after the index date (inclusive of the index date).

^c^*P* values were calculated using the Wilcoxon signed-rank test for continuous variables and McNemar’s test for binary variables.

^d^Examples of other bowel evacuant agents included sodium sulfate-potassium sulfate-magnesium sulfate, polyethylene glycol-potassium chloride-sodium bicarbonate-sodium chloride, and sodium picosulfate-magnesium oxide-anhydrous citric acid.

CIC, chronic idiopathic constipation; SD, standard deviation; SSRI, selective serotonin reuptake inhibitor.

**Table A7.** Number of All-cause and Constipation-related Health-care Visits Before and After Prucalopride Initiation in Patients with CIC Who Previously Used Other CIC Medications

| **Visits** | **Number of all-cause visits**  **(N = 436)**  **Mean (SD), median (range)** | | | **Number of constipation-related visits**  **(N = 436)**  **Mean (SD), median (range)** | | |
| --- | --- | --- | --- | --- | --- | --- |
|  | Baseline period^a^ | Study period^b^ | *P* value^c^ | Baseline period^a^ | Study period^b^ | *P* value^c^ |
| **Outpatient** | 16.95 (15.50),  13.00 (0.00–161.00) | 15.99 (16.43),  11.00 (0.00–157.00) | < .001 | 2.70 (7.56),  2.00 (0.00–149.00) | 1.79 (6.19),  1.00 (0.00–116.00) | < .001 |
| Outpatient office and clinic or urgent care | 15.25 (11.74),  12.00 (0.00–64.00) | 13.77 (11.79),  10.00 (0.00–76.00) | < .001 | 2.29 (2.74),  2.00 (0.00–30.00) | 1.44 (2.83),  1.00 (0.00–33.00) | < .001 |
| Outpatient hospital and surgical center | 0.36 (1.32),  0.00 (0.00–16.00) | 0.26 (1.20),  0.00 (0.00–20.00) | < .05 | 0.05 (0.21),  0.00 (0.00–1.00) | 0.03 (0.18),  0.00 (0.00–2.00) | – |
| Gastroenterologist | 1.53 (1.58),  1.00 (0.00–10.00) | 0.91 (1.42),  0.00 (0.00–13.00) | < .001 | – | – | – |
| Dietitian | 0.09 (0.96),  0.00 (0.00–18.00) | 0.08 (0.76),  0.00 (0.00–11.00) | – | – | – | – |
| Psychiatrist | 0.48 (1.95),  0.00 (0.00–24.00) | 0.48 (2.32),  0.00 (0.00–37.00) | .301 | – | – | – |
| **ER** | 0.47 (1.07),  0.00 (0.00–9.00) | 0.48 (1.01),  0.00 (0.00–7.00) | .560 | 0.07 (0.37),  0.00 (0.00–4.00) | 0.04 (0.21),  0.00 (0.00–2.00) | – |
| **Inpatient** | 0.09 (0.36),  0.00 (0.00–3.00) | 0.11 (0.46),  0.00 (0.00–6.00) | – | 0.03 (0.19),  0.00 (0.00–2.00) | 0.03 (0.19),  0.00 (0.00–2.00) | – |
| Total hospitalization days | 0.89 (5.98),  0.00 (0.00–79.00) | 0.78 (4.07),  0.00 (0.00–50.00) | .883 | 0.25 (1.94),  0.00 (0.00–26.00) | 0.35 (2.61),  0.00 (0.00–41.00) | – |

^a^The baseline period was defined as the 6 months before the index date (the date of the first prescription fill for prucalopride).

^b^The study period was defined as the 6 months after the index date (inclusive of the index date).

^c^*P* values were calculated using the Wilcoxon signed-rank test (significance at 5%).

CIC, chronic idiopathic constipation; ER, emergency room; SD, standard deviation.

**Table A8.** All-cause and Constipation-related Direct Health-care Costs Before and After Prucalopride Initiation in Patients with CIC Who Did Not Have a Capitated Health Plan and Who Previously Used Other CIC Medications (N = 353)

|  | **All-cause**  **Mean (SD), median (range)** | | | **Constipation-related**  **Mean (SD), median (range)** | | |
| --- | --- | --- | --- | --- | --- | --- |
|  | Baseline period^a^ | Study period^b^ | *P* value^c^ | Baseline period^a^ | Study period^b^ | *P* value^c^ |
| **Total** | 16,142 (22,944),  7460  (93–146,322) | 18,331 (30,971),  7769  (289–206,889) | .588 | 1958 (2803),  1313  (0–26,410) | 2664 (3574),  2192  (0–53,399) | < .001 |
| **Pharmacy** | 4595 (8646),  1993  (0–81,903) | 6276 (10,965),  3122  (0–94,589) | < .001 | 982 (1008),  817  (0–6390) | 2010 (1353),  1906  (0–8727) | < .001 |
| **Medical** | 11,547 (20,236),  3913  (0–145,439) | 12,055 (25,677),  2880  (0–185,683) | < .01 | 976 (2620),  174  (0–25,081) | 654 (3390),  51  (0–52,486) | < .001 |
| Outpatient | 7791 (13,790),  3287  (0–145,439) | 8127 (17,534),  2393  (0–152,609) | < .01 | 684 (1634),  157  (0–20,695) | 377 (978),  42  (0–11,421) | < .001 |
| Outpatient other^d^ | 771 (3461),  39  (0–32,237) | 1597 (7511),  21  (0–61,505) | .654 | 85 (1140),  0  (0–20,695) | 39 (612),  0  (0–11,421) | .227 |
| Outpatient hospital and surgical center | 472 (1865),  0  (0–25,668) | 352 (1077),  0  (0–9139) | .611 | 52 (285),  0  (0–2414) | 45 (355),  0  (0–5140) | .324 |
| Outpatient office and clinic or urgent care | 6547 (12,478),  2771  (0–145,439) | 6178 (13,937),  1838  (0–152,609) | < .001 | 547 (1074),  149  (0–6743) | 293 (687),  36  (0–6416) | < .001 |
| Inpatient | 2746 (12,047),  0  (0–107,613) | 2988 (14,536),  0  (0–156,239) | .874 | 145 (1610),  0  (0–24,365) | 245 (2937),  0  (0–45,825) | .740 |
| ER | 1010 (2823),  0  (0–19,890) | 940 (2576),  0  (0–19,586) | .730 | 148 (876),  0  (0–9116) | 33 (259),  0  (0–2728) | < .05 |

All costs are reported in US dollars.

Numbers may not sum to the total costs, owing to rounding and gamma distribution of the data.

^a^The baseline period was defined as the 6 months before the index date (the date of the first prescription fill for prucalopride).

^b^The study period was defined as the 6 months after the index date (inclusive of the index date).

^c^*P* values were calculated using the Wilcoxon signed–rank test (significance at 5%).

^d^Other outpatient costs included costs associated with all other outpatient claims, including costs for laboratory services and home health services.

CIC, chronic idiopathic constipation; ER, emergency room; SD, standard deviation.

**Table A9.** Number of All-cause and Constipation-related Health-care Visits Before and After Prucalopride Initiation in Male Patients with CIC

| **Visits** | **Number of all-cause visits (N = 86)**  **Mean (SD), median (range)** | | | **Number of constipation-related visits (N = 86)**  **Mean (SD), median (range)** | | |
| --- | --- | --- | --- | --- | --- | --- |
|  | Baseline period^a^ | Study period^b^ | *P* value^c^ | Baseline period^a^ | Study period^b^ | *P* value^c^ |
| **Outpatient** | 16.34 (14.99),  12.50 (1.00–97.00) | 14.00 (11.79),  11.00 (0.00–45.00) | .053 | 2.14 (2.12),  1.00 (0.00–10.00) | 1.42 (2.18),  1.00 (0.00–16.00) | < .05 |
| Outpatient office and clinic or urgent care | 14.00 (11.22),  11.00 (1.00–54.00) | 12.37 (10.43),  9.00 (0.00–44.00) | .100 | 2.02 (1.99),  1.00 (0.00–8.00) | 1.37 (2.14),  1.00 (0.00–16.00) | < .05 |
| Outpatient hospital and surgical center | 0.17 (0.44),  0.00 (0.00–2.00) | 0.19 (0.47),  0.00 (0.00–2.00) | – | 0.05 (0.21),  0.00 (0.00–1.00) | 0.01 (0.11),  0.00 (0.00–1.00) | – |
| Gastroenterologist | 1.62 (1.70),  1.00 (0.00–7.00) | 0.93 (1.30),  0.00 (0.00–5.00) | < .01 | – | – | – |
| Dietitian | 0.02 (0.15),  0.00 (0.00–1.00) | 0.01 (0.11),  0.00 (0.00–1.00) | – | – | – | – |
| Psychiatrist | 0.15 (0.52),  0.00 (0.00–3.00) | 0.17 (0.62),  0.00 (0.00–3.00) | – | – | – | – |
| **ER** | 0.52 (0.99),  0.00 (0.00–4.00) | 0.52 (1.21),  0.00 (0.00–6.00) | – | 0.08 (0.35),  0.00 (0.00–2.00) | 0.02 (0.15),  0.00 (0.00–1.00) | – |
| **Inpatient** | 0.22 (0.66),  0.00 (0.00–3.00) | 0.16 (0.48),  0.00 (0.00–3.00) | – | 0.07 (0.30),  0.00 (0.00–2.00) | 0.02 (0.15),  0.00 (0.00–1.00) | – |
| Total hospitalization days | 2.19 (9.27),  0.00 (0.00–70.00) | 1.30 (5.85),  0.00 (0.00–50.00) | – | 0.71 (3.85),  0.00 (0.00–26.00) | 0.38 (2.51),  0.00 (0.00–18.00) | – |

^a^The baseline period was defined as the 6 months before the index date (the date of the first prescription fill for prucalopride).

^b^The study period was defined as the 6 months after the index date (inclusive of the index date).

^c^*P* values were calculated using the Wilcoxon signed-rank test (significance at 5%).

CIC, chronic idiopathic constipation; ER, emergency room; SD, standard deviation.

**Table A10.** All-cause and Constipation-related Direct Health-care Costs Before and After Prucalopride Initiation in Male Patients with CIC Who Did Not Have a Capitated Health Plan (N = 61)

|  | **All-cause**  **Mean (SD), median (range)** | | | **Constipation-related**  **Mean (SD), median (range)** | | |
| --- | --- | --- | --- | --- | --- | --- |
|  | Baseline period^a^ | Study period^b^ | *P* value^c^ | Baseline period^a^ | Study period^b^ | *P* value^c^ |
| **Total** | 23,986 (41,662),  6749  (0–233,021) | 19,256 (32,250),  6696  (330–158,986) | .943 | 2191 (4959),  458  (0–26,410) | 2119 (1864),  1760  (92–8727) | < .01 |
| **Pharmacy** | 5734 (13,637),  1442  (0–81,903) | 7099 (15,449),  2313  (289–94,589) | < .001 | 468 (756),  0  (0–2649) | 1606 (1404),  1305  (0–8727) | < .001 |
| **Medical** | 18,252 (38,049),  3287  (0–231,352) | 12,157 (25,651),  2345  (0–151,682) | < .05 | 1723 (4750),  230  (0–25,793) | 513 (1063),  6  (0–5855) | < .05 |
| Outpatient | 8754 (15,335),  2544  (0–92,955) | 7389 (14,812),  1943  (0–71,207) | < .05 | 775 (1746),  140  (0–9821) | 470 (1034),  6  (0–5855) | .070 |
| Outpatient other^d^ | 2130 (9347),  20  (0–71,794) | 1544 (7891),  0  (0–61,505) | .516 | 108 (691),  0  (0–5356) | 3 (17),  0  (0–103) | .097 |
| Outpatient hospital and surgical center | 196 (617),  0  (0–3178) | 123 (442),  0  (0–2628) | .616 | 44 (271),  0  (0–2027) | 0 (0),  0  (0–0) | .371 |
| Outpatient office and clinic or urgent care | 6427 (9932),  2362  (0–47,590) | 5722 (11,869),  1399  (0–64,398) | .062 | 623 (1261),  124  (0–6743) | 466 (1024),  6  (0–5752) | .128 |
| Inpatient | 7918 (24,741),  0  (0–137,135) | 3823 (15,356),  0  (0–96,710) | .126 | 860 (4505),  0  (0–25,793) | 6 (47),  0  (0–370) | .059 |
| ER | 1580 (3814),  0  (0–17,907) | 945 (2988),  0  (0–14,859) | .179 | 88 (485),  0  (0–3605) | 37 (279),  0  (0–2176) | .529 |

All costs are reported in US dollars.

Numbers may not sum to the total costs, owing to rounding and gamma distribution of the data.

^a^The baseline period was defined as the 6 months before the index date (the date of the first prescription fill for prucalopride).

^b^The study period was defined as the 6 months after the index date (inclusive of the index date).

^c^*P* values were calculated using the Wilcoxon signed-rank test (significance at 5%).

^d^Other outpatient costs included costs associated with all other outpatient claims, including costs for laboratory services and home health services.

CIC, chronic idiopathic constipation; ER, emergency room; SD, standard deviation.

**References**

1. Charlson ME, Pompei P, Ales KL *et al.* A new method of classifying prognostic comorbidity in longitudinal studies: development and validation. *J Chronic Dis* 1987;40:373–383. doi: 10.1016/0021-9681(87)90171-8.

2. Quan H, Sundararajan V, Halfon P *et al.* Coding algorithms for defining comorbidities in ICD-9-CM and ICD-10 administrative data. *Med Care* 2005;43:1130–1139. doi: 10.1097/01.mlr.0000182534.19832.83.
